# The GAP43 gene in neuroblasts is upregulated in response to cell seeding density

**DOI:** 10.17912/micropub.biology.001628

**Published:** 2025-12-19

**Authors:** Mikiya Wakabayashi, Naotaka Nakazawa

**Affiliations:** 1 Graduate School of Science and Engineering, Kindai University, Higashiosaka-city, Osaka, Japan; 2 Faculty of Science and Engineering, Kindai University, Higashiosaka-city, Osaka, Japan

## Abstract

The cell in multicellular organisms are capable of sensing and responding to various types of extracellular stimuli. Recent studies have highlighted the importance of not only biochemical stimuli but also mechanical stimuli in modulating cellular behavior. Mechanical stimuli include substrate stiffness, shear stress, tensile force, and compressive strain, all of which have been implicated in the regulation of gene expression, proliferation, and differentiation. In this study, we investigated the impact of compressive strain by cell seeding density on gene expression in Neuro2A cells, a mouse neuroblastoma-derived neuronal cell line. Specifically, we focused on the expression of Cyclin D1, a well-established proliferation marker, and GAP43, a marker associated with neuronal differentiation. By culturing Neuro2A cells at different cell densities, we found that high cell density upregulated the GAP43 gene but not the Cyclin D1 gene. This result suggests that cell density affects neuronal differentiation through GAP43 gene expression.

**Figure 1. The GAP43 gene is upregulated in response to high cell seeding density:**
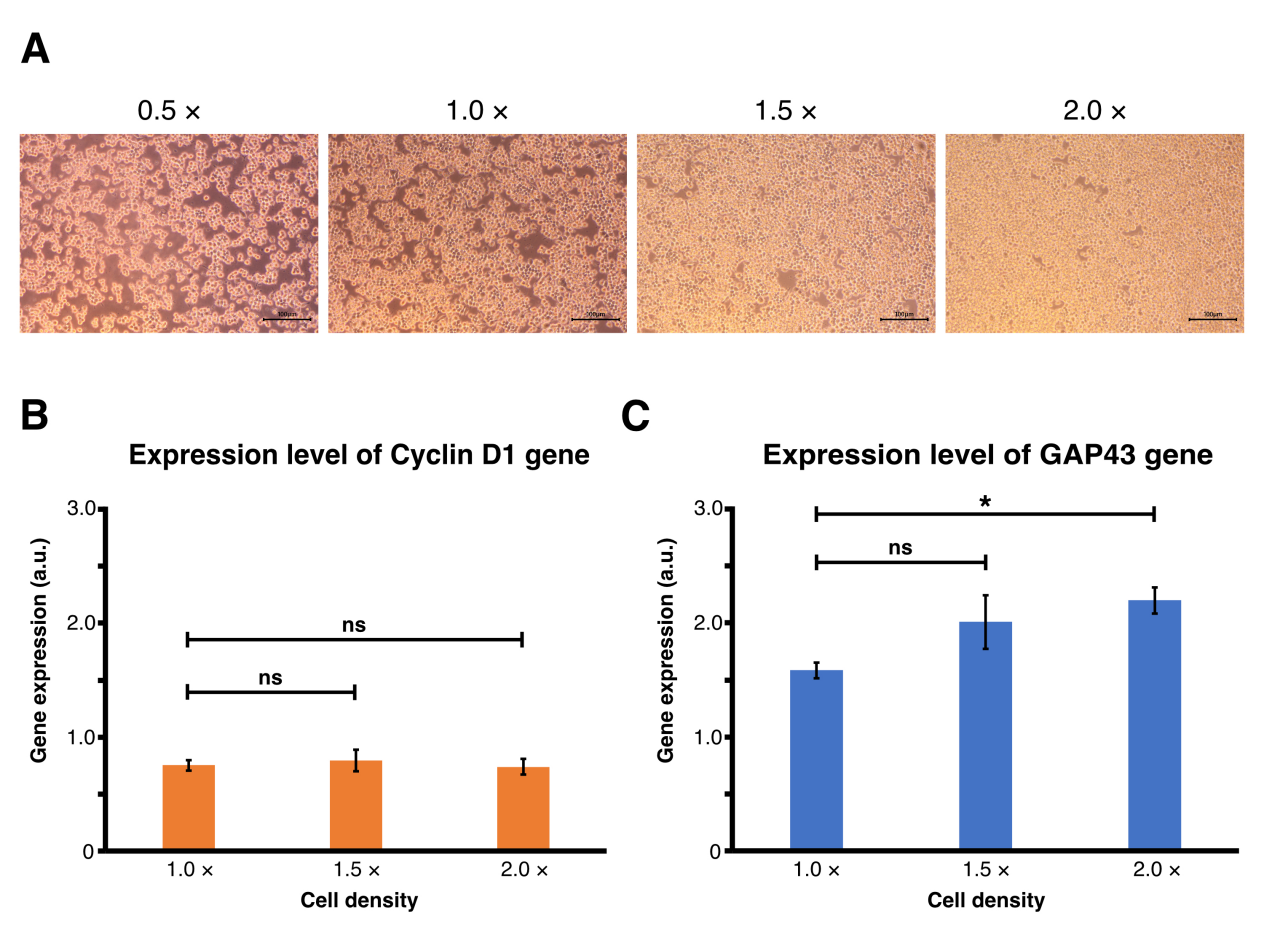
(A) Bright images of Neuro2A cells in the condition of 0.5× density (left), 1.0× density (middle left), 1.5× density (middle right), 2.0× density (right), respectively. Scale bars: 100 μm. (B) A graph showing expression of Cyclin D1 gene relative to the condition of 0.5× density, normalized to expression of the β-actin gene. Each condition was assessed using seven biological replicates. Data in the graph are represented as mean ± SEM. (C) A graph showing expression of the GAP43 gene relative to the condition of 0.5× density, normalized to expression of the β-actin gene. Each condition was assessed using seven biological replicates. Data in the graph are represented as mean ± SEM.

## Description

The cell in our body continuously senses and responds to chemical and mechanical stimuli in its environment. While the influence of soluble biochemical factors such as hormones and growth factors has been extensively studied, recent findings suggest the importance of mechanical cues, such as matrix stiffness, fluid shear stress, and direct compression on cellular behavior including differentiation through the regulation of gene expression (Vogel and Sheetz, 2006; Jaalouk and Lammerding, 2009; Uhler and Shivashankar, 2017; Dupont and Wickström, 2022).

Cell seeding density is critical for cellular conditions in *in vitro* cultures. Due to mechanical properties, high cell density exerts compressive forces on the cells (Faure et al., 2025). Importantly, previous studies suggest that the mechanical influence of cell density or confluence *in vivo* is important for physiological events, such as morphogenesis, brain development, and cell delamination, etc. (Kosodo et al., 2011; Eisenhoffer et al., 2012; Okamoto et al., 2013; Xiong et al., 2020; Lu et al., 2024; Moore Zajic et al., 2025). At the molecular level, cell density is involved in the regulation of gene expression in endothelial cells and mesenchymal stem cells (Heng et al., 2011; Kim et al., 2017). However, the effects of cell seeding density in neuroblast cells have not been widely studied.

In this study, we investigated the effects of cell seeding density on transcriptional regulation for cell proliferation and differentiation in Neuro2A cells, a mouse neuroblastoma-derived cell line. We focused on two representative molecular markers, Cyclin D1, a G1-phase regulatory protein that promotes cell cycle progression from G1 to S phase and maintains an undifferentiated proliferative state (Xiong et al., 1991; Musgrove et al., 2011), and Growth Associated Protein 43 (GAP43), a neuronal protein recognized as a marker of axonal outgrowth and neuronal maturation (Skene and Virág, 1989; el-Husseini and Bredt, 2002). GAP43 is predominantly expressed in postmitotic neurons, where it contributes to cytoskeletal remodeling and membrane expansion during neurite extension and synaptic development (Denny, 2006). To reduce variability in gene expression due to cell cycle heterogeneity, Neuro2A cells were synchronized at the G0/G1 phase by low serum starvation (0.25% FBS) for 96 hours prior to experimental manipulation (Malcolm et al., 2019). Cells were then seeded at four defined densities-0.5× (8.80 × 10 ^5^ cells per 3.5 cm dish), 1.0× (1.76 × 10 ^6^ cells per 3.5 cm dish), 1.5× (2.64 × 10 ^6^ cells per 3.5 cm dish), and 2.0× (3.52 × 10 ^6^ cells per 3.5 cm dish) relative to confluence threshold (1.76 × 10 ^6^ cells per 3.5 cm dish), and cultured in complete medium for 12 hours. Phase-contrast microscopy revealed a progressive increase in cell packing with increasing cell density ( Figure 1A ). In the 0.5× condition, cells were sparsely distributed and rounded with minimal contact. In contrast, cells in the 1.0× condition showed partial alignment and increased intercellular contact, suggesting the onset of mechanical constraint. In addition, cells in the 1.5× and 2.0× conditions were densely packed with indistinct borders and complete monolayer coverage, consistent with increasing degrees of lateral compression. We then performed quantitative real-time PCR on the cells from each condition to assess transcriptional responses to cell density. Gene expression was normalized to β-actin and calculated relative to the 0.5× condition, which was set as the baseline at 1.0. Cyclin D1 expression remained stable across all densities ( Figure 1B ), suggesting that compressive stress from increased crowding does not perturb G1/S regulatory pathways in this context. In contrast, GAP43 expression exhibited a density-dependent increase, with a statistically significant upregulation observed at 2.0× condition compared to 1.0× condition ( Figure 1C ). In the 1.5× condition, although the difference from the 1.0× condition was not significant, the expression level of GAP43 seemed to be gradually increased.

Thus, our results suggest that cell seeding density selectively promotes transcriptional gene expression associated with neuronal maturation, without affecting proliferation-related gene expression. During brain development, neural progenitor cells often encounter fluctuating local densities (Okamoto et al., 2013; Shinoda et al., 2018). Our results support the hypothesis that physical microenvironmental cues, including intercellular compression, are involved in the regulation of gene expression in immature neurons. However, it is difficult to completely exclude biochemical effects on cell differentiation in this experimental setting, as the number of cells in each condition differs. &nbsp;In fact, we could not confirm any clear differences in GAP43 protein levels in the cells grown at different cell densities. In our experimental setting, it is possible that cell density may affect both transcription and translation at different time points. In any case, further research with a distinct experimental setting on mechanical regulation is required to reveal the mechanical impact of cell density on the regulation of gene expression and modulation of cellular functions. The combination of microdevice technology with a primary culture of neural stem cells is a critical approach for revealing the clear effects of cell compression on neuronal cell behavior (Liu et al., 2015; Reversat et al., 2020; Onal et al., 2022; Faure et al., 2025; Nakazawa et al., 2025).

## Methods

**Cell line and culture conditions:** Neuro2A cells were obtained from the JCRB Cell Bank (IFO50081). Cells were cultured in complete medium (D-MEM (High Glucose) with L-Glutamine, Phenol Red and Sodium Pyruvate (Wako, 043-30085) supplemented with 10% (v/v) fetal bovine serum (Corning, 35-079-CV) and 1% penicillin-streptomycin (Nacalai, 936734)) at 37°C in a humidified incubator containing 5% CO₂. To reduce variability in gene expression due to asynchronous cell cycling, cells were synchronized at the G0/G1 phase. Neuro2A cells were first cultured in complete medium until reaching approximately 70% confluency, then switched to low-serum medium (D-MEM with 1% penicillin-streptomycin containing 0.25% FBS) and maintained for 96 hours (Malcolm et al., 2019). After synchronization, cells were reseeded into 3.5 cm dishes (Thermo Scientific, 12-9971) at four different densities: 0.5× (8.80 × 10 ^5^ cells per dish), 1.0× (1.76 × 10 ^6^ cells per dish), 1.5× (2.64 × 10 ^6^ cells per dish), and 2.0× (3.52 × 10 ^6^ cells per dish), based on the experimentally determined confluency threshold of 1.76 × 10 ^6^ cells per dish. All cultures were incubated for 12 hours in complete medium before RNA extraction.

**Phase-contrast imaging:** Morphological observations were performed using IX71 with x10 lens (NA: 0.30) (Olympus) and WRAYCAM-NOA2000 (WRAYMER). Images were acquired from cultures at each density condition after 12 hours of incubation. Image processing was performed by Microstudio (WRAYMER).

**Quantitative real-time PCR:** Total RNA was extracted using RNA Premium Kit (Fastgene, FG-81050) according to the manufacturer’s instructions. RNA samples were reverse-transcribed into cDNA using Scriptase II cDNA 5x ReadyMix (Fastgene, NE-LS65). Quantitative PCR was performed using KAPA SYBR Fast qPCR (KAPA BIOSYSTEMS, KK4601) on a Step One Plus Real-Time PCR System (Applied Biosystems). The following gene-specific primers were used; β-actin (forward: 5’-GTACCACCATGTACCC-3’, reverse: 5’-AACGCAGCTCAGTAAC-3’), CyclinD1(forward: 5’-TTCAGGGAGGAAATGG-3’, reverse: 5’-CCCATGCTGTCACTCT-3’), GAP43(forward: 5’-GGCTCTGCTACTACCG-3’, reverse: 5’-GGCTTGTTTAGGCTCC-3’). Gene expression levels were normalized to β-actin and calculated using the ΔΔCt method. All experiments were performed in triplicate.

**Statistical analysis:** Data were analyzed using Excel (Microsoft) and Python 3.0. Statistical comparisons were performed using Dunnett’s multiple comparison test, with the 1.0× condition as the control. Differences were considered statistically significant at *p < 0.05.
